# Removal of Right Half of Lower Jaw by Slow Enucleation

**Published:** 1887-11

**Authors:** 


### SELECTIONS.

### Removal of Right Half of Lower Jaw by Slow Enucleation.

Dr. Joseph W. Howe sent a patient to the New York Pathological Society (June 8, 1887), with the following history: Thomas R., 23 years of age, was admitted to St. Francis Hospital with the following symptoms: On October 4 he received a severe blow on the under surface of the chin, which exposed the bone. The wound was sewed up at St. Luke's Hospital, but failed to unite. The jaw afterward became very much swollen and painful, and he sought admission to St. Francis Hospital. Dr. Howe made an examination in the early part of November, and found an extensive swelling extending from the symphysis to the posterior part of the ramus of the jaw on the right side, and a small hole running up under the chin behind the jaw, which led to the dead bone. The teeth on the affected side were loosened, and the alveolar process stripped bare and rough and bathed in pus. Pressure on the outside, over the jaw, forced a quantity of pus out, both from between the periosteum and bone and external to the periosteum. The periosteum was separated along the upper line of the jaw back to the ramus, and in some parts had been completely destroyed. The lower teeth were removed from the jaw the following day, and a portion of the periosteum separated. This process of separating the periosteum from the bone was repeated at intervals for several weeks, while the parts were thoroughly cleaned and the patient's general health was kept up by tonics and stimulants. With the stripping of the bone, the periosteum gradually thickened so as to increase the swelling on the affected side. Three weeks previous to the first operation, he made a careful examination under ether, and separated the greater portion of the periosteum and other tissues from the bone. Pieces of lint steeped in carbolic acid and glycerine were placed on each side of the dead jaw, and the patient was allowed to return to the ward. More swelling and fever followed the operation. On the following Monday he completed the operation, removing the last attachment at the symphysis and pulling out the diseased jaw, leaving behind a good basis for a new jaw in the ossifying periosteum. The slow enucleation of a diseased jaw is a comparatively simple process; very little force and very little cutting are required to remove it. The patient has now a new jaw, which has grown from the old periosteum.—*Medical Record*, August 20, 1887.

